# HCN1-mediated interactions of ketamine and propofol in a mean field model of the EEG

**DOI:** 10.1186/1471-2202-14-S1-O22

**Published:** 2013-07-08

**Authors:** Ingo Bojak, Harry C Day, David T J Liley

**Affiliations:** 1 School of Systems Engineering, University of Reading, Whiteknights, Berkshire, RG6 6AY, UK; 2 School of Psychology (CNCR), University of Birmingham, Edgbaston, Birmingham B15 2TT, UK; 3 Donders Institute, Radboud University Nijmegen (Medical Centre), 6500 HB Nijmegen, The Netherlands; 4 Brain & Psychological Sciences Research Centre, Swinburne Uni. of Tech., Hawthorn, Victoria 3122, Australia; 5 Cortical Dynamics Ltd., Suite 4, 462 Burwood Road, Hawthorn, Victoria 3122, Australia

## 

Ketamine and propofol, two popular anesthetic agents, are generally believed to operate via disparate primary mechanisms: ketamine through NMDA antagonism and propofol through the potentiation of GABA_A_-gated receptor currents. However, surprisingly the effect of ketamine on the EEG is markedly altered in the presence of propofol. Specifically, while ketamine alone results in a downshift of the peak frequency of the alpha rhythm, and propofol keeps it roughly constant - when administered together, they increase the alpha peak frequency [1].

Recently it has been found that both ketamine and propofol inhibit the hyperpolarization-activated cyclic nucleotide-gated potassium channel form 1 (HCN1) subunits, which induces neuronal membrane hyperpolarization [2]. Furthermore, HCN1 knockout mice are significantly less susceptible to hypnosis with these agents; but equally affected by HCN1-neutral etomidate [2].

**Figure 1:**
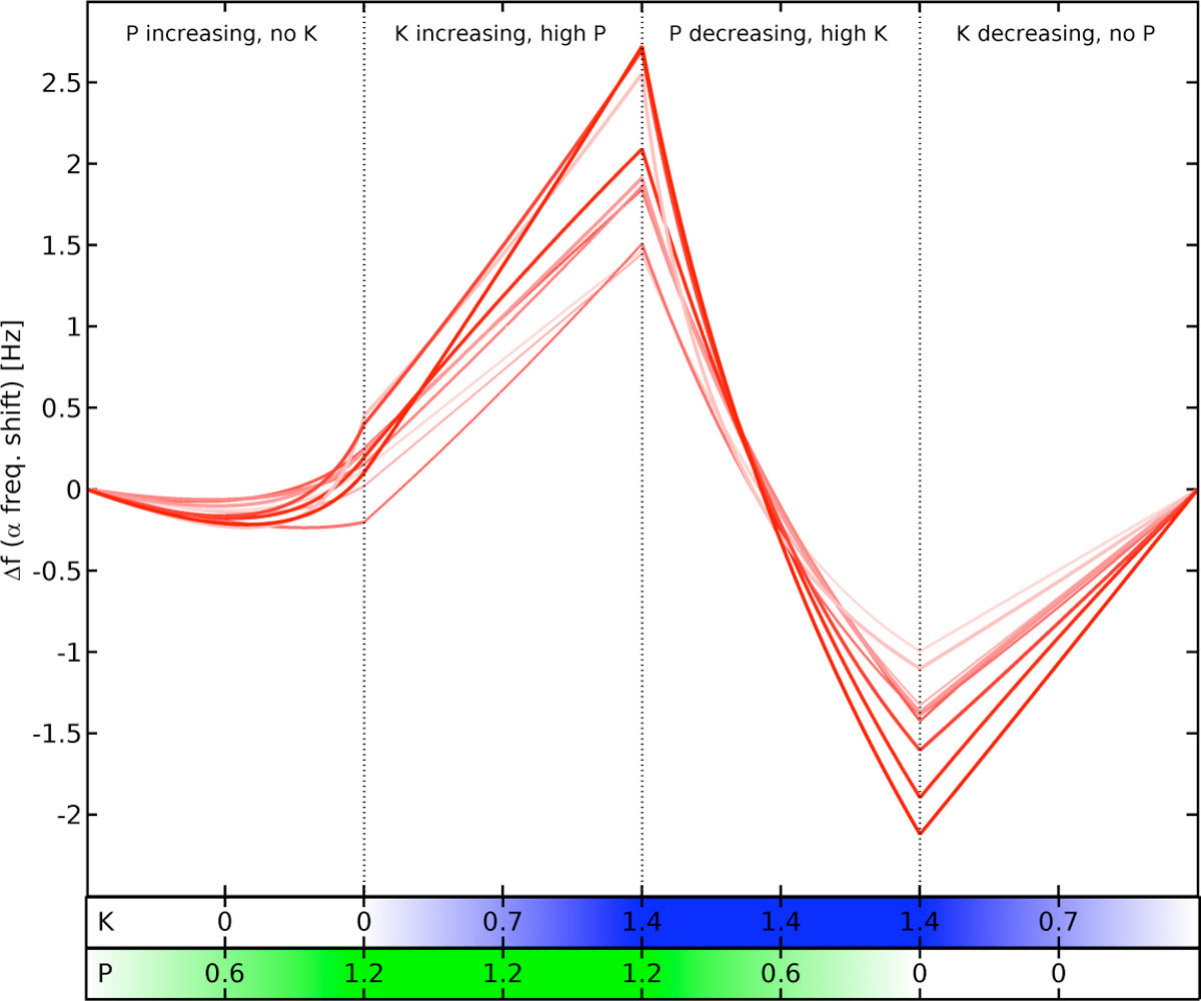
**Predicted shift of the alpha peak frequency of ten parameter sets during four phases of linear change to the normalized ketamine (K) and propofol (P) concentrations, respectively**.

We show here [3] that an established mean field model of electrocortical activity can predict the EEG changes induced by combining ketamine and propofol by taking into account merely the HCN1-mediated hyperpolarisations, but neglecting their supposed main mechanisms of action (NMDA and GABA_A_, respectively). See Figure 1.

Our results suggest that ketamine and propofol are infra-additive in their HCN1-mediated actions. This is consistent with independent experimental evidence[4]. We show here that the HCN1-mediated actions of ketamine and propofol, hitherto neglected by models of anaesthetic action, can not only explain a range of counterintuitive induced EEG changes but also predicts the infra-additivity of these drugs.
